# 

**Published:** 1885-04

**Authors:** 


### CONTENTS.

PROCEEDINGS.

Mississippi Valley Association of Dental Surgeons ........................161

SELECTIONS.

Hurry and Worry........................................................................193
The Relations between the Teeth and the Womb.........................194
Sleeplessness ..........................................................................195
Rheumatic Glossitis....................................................................196
The Gastroscope........................................................................197
About Gray Hair..........................................................................198
Running for Trains......................................................................199
Chlorine, Bromine, and Iodine as Disinfectants...........................199
Insects and Flowers....................................................................200
Menthol.......................................................................................200
Excision of the Superior Maxilla..................................................201
Peroxide of Hydrogen.................................................................202
CORRESPONDENCE......................................................................203
SALMAGUNDI..............................................................................203

EDITORIAL.

Dental Law..................................................................................208
Illinois State Dental Society........................................................212
Mad River Valley Dental Society.................................................212

